# Making Polyol Gummies by 3D Printing: Effect of Polyols on 3D Printing Characteristics

**DOI:** 10.3390/foods11060874

**Published:** 2022-03-18

**Authors:** Hao Le, Xiaorui Wang, Yabo Wei, Yunfeng Zhao, Jian Zhang, Lianfu Zhang

**Affiliations:** 1 Food College, Shihezi University, Shihezi 832003, China; 20192111005@stu.shzu.edu.cn (H.L.); wangxiaorui0923@163.com (X.W.); 18935813163@163.com (Y.W.); yunfeng@shzu.com.edu.cn (Y.Z.); 2 School of Food Science and Technology, Jiangnan University, Wuxi 214122, China

**Keywords:** 3D printing, gummy, polyols, rheological properties, printing performance

## Abstract

With growth of confectionery industry, there is a great demand for candy shape, and 3D printing technology is way to achieve it. The printing properties of gummy, which is formed of gelatin and low acyl gellan as gel, maltol, erythritol, sorbitol, and xylitol as sweeteners, were tested in this study. Gummies’ rheological properties, 3D printing properties, and textural qualities were measured using a rheometer, FTIR, and SEM in this study. The strength of the hydrogen bonds will be affected by the addition of polyol, after which the excluded volume effect of polyol and viscosity will become the most important aspect. Polyols increased the gelation temperature (Tgelation), improved the gel network, and improved hydrogen bonding in the gel, according to the findings. Yield stress, shear recovery performance, and gel strength were initially increased, then decreased, when polyol concentration was increased. It had a 40.59 °C gelation temperature, an 82.99% recovery rate, noticeable shear thinning features, high self-supporting performance, and textural qualities when ink with 35 g maltitol and 30 g erythritol gave the best printing performance. This research serves as a foundation for the development of individualized, bespoke 3D printed gummies in the future.

## 1. Introduction

The confectionery industry is one of the main food industries worldwide and has a market value of approximately USD 82.3 billion according to recent years. The expected growth of this market is 3.6% annually [1]. Jellies and gummies are a popular and significant growing candy category in the candy market [2]. The traditional manufacturing of jellies and gummies generally follow the same steps of (i) ingredient mixing; (ii) cooking; (iii) cooling the syrup and adding food additives; (iv) syrup dosing in dried starch powder molds; (v) curing or stoving; (vi) finishing [3]. Traditional manufacturing is gradually hard to meet the needs of consumers for the shape of gummies. To produce different shapes of gummies, molds are widely used in some creative soft candy, which requires significant human input, denser manual handling during plating, more storage and more use of plastics [4]. There is a great demand for the new production method to meet personalized products.

The technology of 3D printing, also known as additive manufacturing, an emerging technology in recent years, has illustrated many advantages such as the personalization of nutrition, customization design of food texture, and decrease of human input dependency. This would possibly provide the confectionery industry with a new feasible solution [5,6]. As a result, 3D printing technology has gotten a lot of attention as technology has progressed. It can ideally match food processing because of the rapid prototyping and diversity of 3D printing technology. As a result, in recent years, the development and application of food 3D printing technology have become a research priority. The current study on food 3D printing technology is mostly focused on new food materials, such as lemon juice gel, cheese, pectin gel [7,8]. The correlation between gel rheological properties and 3D printability and the synergistic effect of polyol on the gelation mechanism of hydrocolloids have been partially studied. To our knowledge, currently, the information focused on this subject is still quite lacking. The gummy and marshmallow candy gels are complicated gel systems. Although sugars are not part of the gel network, they can considerably aid in the development of gels and candy [9]. Sugars and polyols have been discovered to improve the network strength and thermal durability of gelatin gels [10,11,12]. However soft candy gel’s rheological, structural, and textural properties are yet unknown and 3D printability is significantly influenced by rheological characteristics and material structure [13,14]. The development and application of 3D printing fudge technology can increase the value of gummies by increasing the number of applications for 3D printing food with a personalized and detailed shape, improving the value-adding to artistry in edibles, reducing the need for molds and experienced workers, and increasing the value of gummies [15]. However, 3D printers cannot manage larger capacity (mass production) in a short period of time, and the most significant issue is that there are fewer appropriate materials; material performance must meet the standards of 3D printing, which is currently understudied. So studying the 3D printing characteristics of gummies is important for the confectionery industry.

The impact of polyols on the development of easy-to-use customized gummies via 3D printing is one of the goals of this study. For the reason, products widely used in the confectionery industry were chosen as their basis, namely Gelatin and low acyl gellan.

Gelatin is a high molecular weight polypeptide with good water solubility and has a triple helix structure [16]. Gellan is produced by *Sphingomonas elodea*. The structure of deacylated gellan is formed by repeated polymerization of basic units composed of four monosaccharide molecules and a negatively charged COO- group on glucuronic acid [17]. They are widely used in various kinds of candy, including soft candies, marshmallows, fruit juice gels, and so on [18]. The combination of gelatin and low acyl gellan has been found in studies to give optimal texture or protein–polysaccharide interaction [19]. According to Papageorgiou, the physical properties of low acyl gellan and gelatin gel were strengthened synergistically, resulting in improved hardness, gelation rate, and gel temperature [20]. As the main ingredient of candy, sugar is widely used in the candy production industry. Sugar can improve the rheological properties of gels, raise the temperature of gels, and aid in the creation of gels [11,21]. Sugar processing, on the other hand, needs greater temperatures and is more prone to crystallization, both of which are detrimental to 3D printing. Obesity and diabetes are on the rise around the world, thanks in part to the excessive use of sweets such as sucrose [22]. Polyols have limited absorption compared to sugars and deliver fewer calories and blood glucose, making them an excellent option for traditional sweeteners [23], meanwhile they are more stable and not easy to crystallize. As a sucrose alternative, erythritol, sorbitol, xylitol, and maltol have been widely used to confectionery industry. However, the impact of polyols on polysaccharide gels has received less attention.

Even though polyols have been proved to be a healthy sweetener, little research has been done on their use as additives in 3D printing inks. As a result, the research on the use of polyols as additives for 3D printing inks is unique; 3D printing technology is a hot topic right now, but its application in the food business is still limited, with little research on the technology’s application in soft candy. As a result, it makes sense to use 3D printing technology to create fudge. In order to explore the effect of polyols on gel 3D printing characteristics, gelatin and low acyl gellan gum are used as hydrocolloids (gelling agents), and erythritol, sorbitol, xylitol, and maltol are added as sweeteners. The 3D printing features of gummy gel and a combination of maltitol, sorbitol, and xylitol were investigated using rheological analysis, printing adaptability evaluation, and texture analysis. As a result, this study could be useful for 3D printing polyol gummies.

## 2. Materials and Methods

### 2.1. Materials

Food grade gelatin (260 freezing power), low acyl gellan, erythritol (ery), and sorbitol were purchased from Yinuo Biotechnology Co., Ltd., Zhejiang, China. Liquid maltitol (mal) was purchased from Tianli Pharmaceutical Co., Ltd., Shandong, China Xylitol was purchased from Silicon Valley Technology Development Co., Ltd., Tianjin, China. Food grade polyglycerol ricinol ester was purchased from Qingyuan food additives Co., Ltd., Beijing, China. Food grade hydrocolloids and polyols do not contain fillers, preservatives, expansion agents, and spices.

### 2.2. Food Ink Preparation

Liquid maltitol is a sweetener that has high sweetness and low toxicity and has properties that are quite comparable to sugar. In terms of production and research, maltitol has been recognized as the default sweetener. The effects of various maltitol concentrations, as well as the effects of other polyols, were examined. Prepare ink according to Table 1. We took 14 g gelatin, add 60 mL of water, dissolvef at 80 °C, added 1.4 g low acyl gellan, stirred and dispersed evenly, and kept it warm for standby. The polyols were mixed evenly according to the formula (iKA-T25) at 95 °C. The gel was poured into polyols and continued to stir. The ink was lowered at high temperatures, and a large number of bubbles were generated. The polyglycerol ricinoleic acid ester was used to defoam. When the stirring rate did not change significantly, the stirring was finally stopped, and the final water content of the ink was weighed.

### 2.3. Scanning Electron Microscopy (SEM)

The low concentration polyol gel mixture was prepared according to Table 2. The effect of polyols on the gel was studied by SEM (su8010, Hitachi Co., Tokyo Japan). According to Pant [24] a small quantity of ink was spread on the Petri dish, and kept at −20 °C for 12 h, then freeze-drying in vacuum conditions for 24 h. The dried samples were then mounted on circular stubs with double sided adhesive tapes and sputter coated with gold, the microstructure of the sample was observed at 1000×.

### 2.4. FTIR

The low concentration polyol hydrocolloid mixture was freeze-dried and mixed with KBr powder (1:100, *w*/*w*). The FTIR Molecules spectrum of low concentration polyol hydrocolloid mixture was recorded with transmittance at 4000–400 cm^−1^ on Bruker Vertex 70V FTIR spectrometer (Bruker, Karlsruhe, Germany).

### 2.5. 3D Printing of Food Inks

An extrusion-based food in a 3D food printer (Time Printing Technology Co., Ltd., Hangzhou, China) was used to print different 3D samples. For the experiments, cylinders 15 mm in diameter by 15 mm in height, cubes with 10 mm sides and hollow cylinders 2 mm in width and 15 mm in height were printed, respectively. The printing parameters were fixed as follows: the nozzle size was 1.2 mm, the printing temperature was 75 °C, the printing speed was 15 mm/s, the filling was 100%, and the layer height was 1.15 mm. The title setting lithography (STL) model was designed by Autodesk 123d and sliced by slic3r.

### 2.6. Rheological Characterization

The rheological properties of the gel system were measured by rheometer (Anton Paar MC302, Graz, Austria) with a plate–plate geometry (20 mm diameter, 0.1 mm gap) and the extra samples were extruded and removed to prevent the edge effect. A thin layer of silicone oil was applied around the fixture to prevent moisture evaporation. The sample was heated to 75 °C in advance and equilibrated at the initial measurement temperature for several minutes to reach a stable state. Firstly, the linear viscoelastic region was measured by oscillatory scanning. The static rheological test was conducted in the flow ramp model with the shear rate increasing from 0.01 to 100 1/s at 75 °C, the viscosity and stress of the samples were measured respectively. In the stress scanning test, the frequency was set to 1 Hz and the stress was 1–1000 Pa. the elastic modulus (G′) and viscous modulus (G″) of the sample at 75 °C are measured, and the intersection was defined as the yield stress [25]. Temperature ramps were performed at 1 s^−1^ from 80 °C to 25 °C with a cooling rate of 1 °C/min. The gelation temperature (Tgelation) was calculated by extrapolating the high and low-temperature asymptotes of the viscosity and specifying the temperature at which these intersects [26]. The shear recovery characteristics of the gels were determined by a 3ITT thixotropic test at 75 °C, and low speed shear for 180 s with 1 s^−1^, then high speed shear for 90 s with 100 s^−1^, and low speed shear for 180 s. The shear recovery characteristics of the system were characterized by the ratio of the apparent viscosity of the system at the first 30 s of the third stage to the average apparent viscosity of the first stage [24]. In addition, the dynamic rheology was tested at 25 °C, the strain was 0.1% (in the linear viscoelastic region), the angular frequency was 0.1~100 rad/s, and the complex modulus G* of the gel was calculated to define G* (G*, G* = (G′2 + G″2) 0.5), and all data were tested three times.

### 2.7. Textural Properties Characterization

TA XT plus (Stable Micro System, Surrey, UK) was used for double cycle test to obtain force-time curves. Print the food inks with different formulas into cylinders with a diameter of 15 mm and a height of 15 mm. The test conditions are as follows: using P36R probe, the speed before and after the test was 1 mm/s, the compression rate was 45%, the trigger force was 5 g and the residence time was 5 s, repeated 6–8 times for each sample.

### 2.8. Data Analysis

The statistical software SPSS was used to analyze the data, and origin 2021b was used to draw the chart. Using Duncan test, significance was denoted on top of specific columns the difference of *p* < 0.05 was considered to be significant.

## 3. Results

### 3.1. 3D Printing Performance

**Figure 1:**
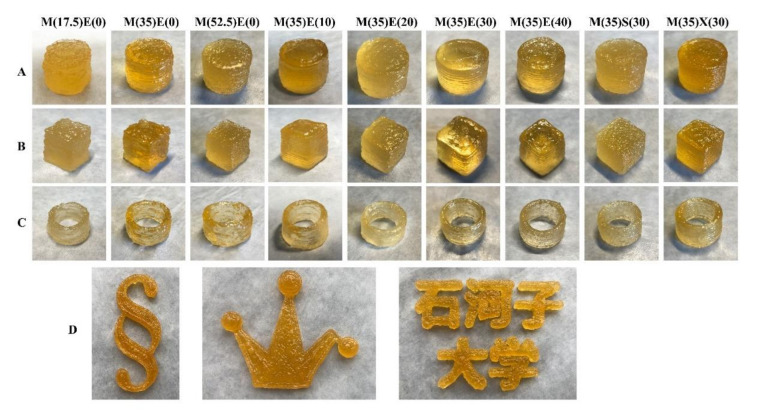
Printing performance with different formulation inks. (**A**) Solid cylinders with diameter of 15 mm and height of 15 mm. (**B**) Cubes with side length of 10 mm. (**C**) Hollow cylinders with wall thickness of 2 mm and height of 15 mm. (**D**) Gummies of different shapes printed with gummy ink.

Figure 1A shows the solid cylinders with a diameter of 15 mm and height of 15 mm, manifested the flatness of the multilayer stacking (extrusion fluency of the ink). The gummy print model becomes easier to shape as the amount of maltitol applied grows from 17.5 g to 52.5 g, as shown in the figure. The cause of this occurrence may be due to the addition of polyols; the viscosity of ink system is gradually affected by the characteristics of polyols [27]. Ink will gradually behave like polyols, which will reduce viscosity at high temperature, and its -OH will enhance gel network and improve printing performance [28,29]. When erythritol is added to the printing material on a regular basis, the surface smoothness improves dramatically. The surface smoothness appears to be gradually smooth as erythritol is added from 0 to 40 g, when 30 g erythritol is added, the cylinder’s surface is relatively smooth, the boundary of each layer of stacking is very clear and smooth, and the bottom of the cylinder did not increase, but 40 g erythritol began to show partial bottom adiposity. This may be due to hydrogen bonds between polyols being unable to form a stable gel, thereby weakening the gel strength [30,31]. Considering the effect of maltitol on stomach and the improvement of printing effect is less than erythritol, we believe that when the amount of maltitol added is 35 g, the amount of erythritol added is 30 g, which has the optimum effect.

Figure 1B depicts a cube printed in the experiment with a side length of 10 mm, demonstrating the circumstance of right-angle printing (the situation where fine objects were printed and reduced). Right-angle printing has higher requirements on the printing material since the printing material was prone to deviation when generating a right angle during right-angle printing. The printed soft candy structure continues to increase as the amount of maltitol added increases from 17.5 g to 52.5 g, as seen in the graph. The surface smoothness revealed a steady smoothness as the amount of erythritol added rose from 0 g to 40 g. This was the same reason as previously discussed. However, it is easy to see that right-angle printing still has a significant issue when printing soft candy cubes.

Figure 1C shows the hollow cylinders with a wall thickness of 2 mm. Solid cylinders with a diameter of 15 mm and height of 15 mm manifested the supportability of the material, and the printed gummy structure becomes more even as the amount of maltitol added increases from 17.5 g to 52.5 g. The surface smoothness revealed a steady smoothness as the amount of erythritol added rose from 0 g to 40 g.

Figure 1D shows soft candies with different shapes printed by inks with 30 g erythritol. The restoration accuracy of Chinese characters with sharp angles is poor, but the accuracy of crown and music symbols are high because they are made up of curves or large angle lines. These shapes show the excellent personalized customization performance of 3D printing soft candies.

Sorbitol and xylitol inks with this ratio have different printing performance from to erythritol. The proportion of inks is related to the types of polyols. We selected some of them for rheological analysis to explore the impact of polyols on printing performance.

### 3.2. Effect of Polyols on Temperature Characteristics of Ink

Rheological properties are often used to evaluate the printing characteristics of materials; the thermal response behaviors of inks with different polyol contents were evaluated by rheological tests. In 3D printing, the viscosity of temperature sensitive ink will change greatly with the difference of temperature, which means that paying attention to the relationship between temperature and viscosity is very important for 3D printing [32,33].

**Figure 2:**
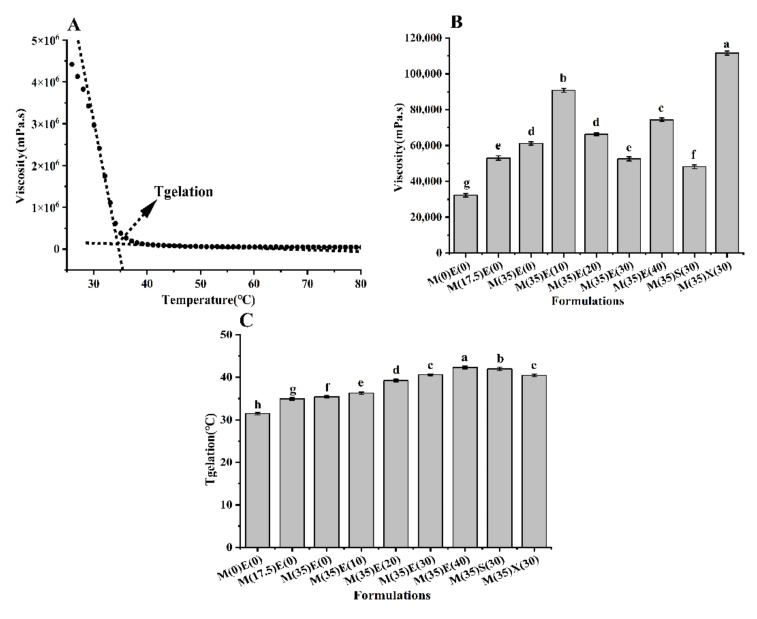
(**A**) Illustrative example of gelation temperature (Tgelation) for the composition M(17.5)E(0) with a cooling rate of 1 °C/min. Tgelation was determined by extrapolating the high and low temperature asymptotes of the viscosity and specifying the temperature at which these intersect. (**B**) Viscosity of different gummy inks at 75 °C. (**C**) Tgelation of inks with different polyol formulations. Different lowercase letters denote significant differences (*p* < 0.05).

Temperature sensitivity was evident in all formulations (Figure 2A, Figures S1 and S2). At temperatures over 35 °C, the viscosity of all inks reduced rapidly as the structure of the helical entangled gel network began to break down, the gel network was destroyed, and polyols were liberated from the gel network [11,20,21]. Figure 2B shows the viscosity with different gummy ink in printing temperatures—the viscosity increased as the maltitol content increases. Following adding 10 g of erythritol, the viscosity increased to its peak value, after which the viscosity dropped then increased as the erythritol concentration increased. The viscosity of gummy ink contained 30 g erythritol was close to the viscosity of gummy ink containing only 17.5 g maltitol. Sorbitol and xylitol have different temperature sensitive properties; sorbitol has a lower viscosity than xylitol.

The relationship between temperature and viscosity is seen in Figure 2A; the Tgelation was recorded by extrapolating the high temperature and low-temperature asymptote of viscosity and specifying the temperature at which they intersect to the record [26]. Polyols were found to have a significant impact on gelation, which is consistent with previous research [30] (Figure 2C). The gel temperature increased considerably (*p* < 0.05) with an increasing polyol, from about 30.44 ± 0.28 to 42.33 ± 0.32 °C, and the gel temperature of xylitol ink was about 40.45 ± 0.3 °C, which was close to erythritol ink (around 40.59 ± 0.2 °C). The gel temperature of sorbitol was higher (about 41.93 ± 0.4 °C).

The ink included more water when the polyol concentration was low, and the gel reacted with the water first, causing phase separation. Despite the lower viscosity, the printing effect was poor. They printed identically because the viscosity of the ink containing 30 g erythritol and xylitol was similar. As sorbitol inks have a higher viscosity than erythritol inks, they have a lower printing performance.

The gel temperature of the 3D printing ink has an impact on the properties of the ink. Pageorgiou discovered that adding sugars such as sucrose to the gel material can hydrogen-bind the substance and improve the gel structure. Additionally, when the sugar level of the gel increases, the gel’s performance improves [11,20,21]. Sorbitol had a higher gel temperature than xylitol and erythritol in this investigation, which could be due to its lower solubility than other polyols, which enhances hydration and local gel concentration, and affects the change in gel temperature.

The addition of polyols changed the temperature and viscosity of the gel, and the temperature and viscosity of the gel determined the rheology of the gel, according to this study. The addition of polyols had a considerable impact on the colloid’s rheological properties. We also discovered that the viscosity of the ink will considerably rise when the ink cools near the nozzle. Additionally, 3D printers with superior temperature equipment are required.

### 3.3. Effect of Polyols on Yield Stress of Inks

The extrusion process in 3D printing was easier for ink with low yield stress [34]. The yield stress also reflects the mechanical strength of the ink, which after printing, was needed to support subsequent stacking layers [35,36].

**Figure 3:**
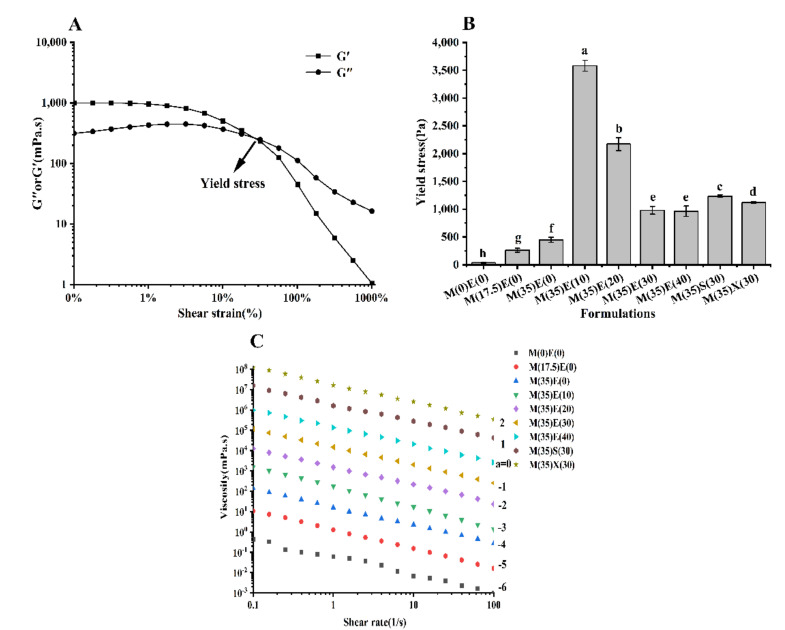
(**A**) Illustrative example of yield stress for the composition M(17.5)E(0) at 75 °C. The yield stress of inks has been determined as the cross-over point where G′ equals to G″. (**B**) Yield stress of inks with different polyol formulations at 75 °C. (**C**) Viscosity for different inks at temperature of 75 °C over shear rate of 0.01–100 1/s. Different lowercase letters denote significant differences (*p* < 0.05).

The oscillation stress sweep mode test was carried out to determine the yield stress of food ink. With the increase of shear stress, the microstructure of ink collapsed and began to flow. Therefore, the yield stress of ink was determined as the intersection of G′ and G″ (Figure 3A) [25]. As shown in Figure 3B, when the ink did not contain polyols, the yield stress was low, the yield stress of 35 g maltitol increased by 67% (from 264.5 Pa to 443.83 Pa) compared with 17.5 g maltitol. Compared with other formulations, the yield stress of 10 g erythritol increased significantly (*p* < 0.05). First, the water content of the ink decreased, and the polyol ratio increased, resulting in an increase in the viscosity of the ink. The gel network strengthened and the yield stress increased when polyols were added to the gel compared to the gel without polyols [30,31,37]. The hydrophilicity of polyols produces a spatial repulsion effect, reduces the water that the colloid can contact, increases the gelatin concentration of low acyl gellan, increases the aggregation rate of both, strengthens the crosslinking, and increases the yield stress. Liu et al. also found that the addition of potato starch will improve the viscosity and yield stress of the mixed system of carrageenan and xanthan gum [38]. The interaction between polyols, polyols and gels is enhanced with the increase of polyol content, due to the hydrogen bonds between polyols cannot form stable gel, thereby weakening the gel strength [30,31]. The yield dress of 30 g erythritol (980.5 mPa·s) is significantly higher than that of 40 g erythritol (945.86 mPa·s). Sorbitol and xylitol possess more hydroxyl groups and make more hydrogen bonds with the gel, and their gelation performance is better than that of erythritol. So its yield stress is also relatively higher, which is consistent with its worse printing performance.

### 3.4. Effect of Polyols on Shear-Thinning Behavior of Inks

After overcoming the yield stress, the pressure required to maintain the continuous flow of ink will depend on their viscosity and shear thinning behavior. Hydrocolloids are widely used in the food industry, which can be added to food to change the rheological properties of food [38]. As shown in the Figure 3C, all samples of soft candy ink showed a shear thinning behavior (the data have been shifted vertically by a factor of 10^a^ to reduce over-lapping). The viscosity of the ink decreased with the shear rate increase from 0.1 to 100 1/s. This is due to the formation of intermolecular aggregates between the gel and polyols in the ink, mainly by hydrogen bonding and polymer entanglement [39]. The aggregates have higher viscosity at low shear rates. The structure is destroyed rapidly at high shear rate. The gel system displayed shear-thinning behavior and low viscosity when the polyols were not added; however, the inclusion of polyols does not modify the shear-thinning behavior but does affect the viscosity of the ink, which is related to the gel formation [38,40,41]. With the addition of polyols, the change of viscosities in all inks were similar with the trend of yield stress at low shear rates, which reached a maximum after adding 10 g erythritol and then gradually decreased, this result indicated that excessive polyols would prevent the gel chains from aggregating to form gels [42]. At high shear rate, the viscosity increases with the increase of polyol content. The ink with only 35 g of maltitol is highly viscous at this water content, which may be a balance of water, gel, and polyol content. The viscosity characteristics of polyol itself will have a great impact on the viscosity characteristics of the final ink. The viscosity of sorbitol and xylitol is higher than erythritol and can provide more hydrogen bond groups, which makes the initial viscosity and the viscosity at high shear rate of adding sorbitol and xylitol ink are higher than erythritol ink. The ink added with 30 g erythritol has appropriate viscosity at low shear rate and high shear rate, which makes its printing performance better.

### 3.5. Effect of Polyols on Shear Recovery Behavior of Inks

**Figure 4:**
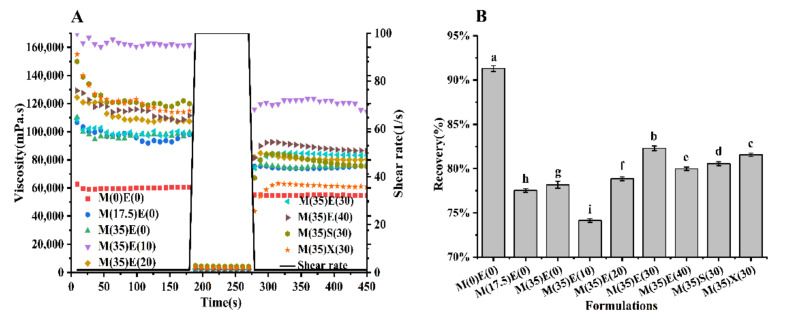
(**A**) Recovery tests of inks conducted at 75 °C under alteration of high (100 1/s) and low (1 1/s) shear rate. (**B**) Shear recoverability of inks determined as the percentage of viscosity obtained during the first 30 s in the third step after high shear rate (100 1/s) based on the average viscosity obtained in the first step. Different lowercase letters denote significant differences (*p* < 0.05).

In the printing process, when the ink is extruded from the nozzle tip, the shear force first decreases, then increases, and finally decreases. Thixotropic inks are ideal for extrusion printing [43]. Figure 4A shows the recovery behavior of ink viscosity after the change of shear rate. The viscosity of all inks decreases significantly at high shear rate, recovers rapidly at low shear rate, and reaches a stable state rapidly in 30 s. The recovery rate of all inks containing polyols was between 75 and 82%, and the recovery rate of ink with 30 g erythritol was the highest (82.29%), but was lower than the ink without polyol (91.45%). The inclusion of polyols enhanced the viscosity of bulk water surrounding the polyols molecules in the continuous phase as compared to the gel without polyols. As a result, in gelatin–water–sugar systems, the thermal motion of stabilized water diminishes [44,45], the ink recovery time was lengthened, and the recovery rate was reduced. After adding polyol, the recovery rate of ink with only maltitol increases slightly. After adding erythritol, the recovery rate decreases first and then increases. The addition of a small amount of erythritol (10 g) increased the number of hydrogen bonds in the fudge ink and decreased the water content of the ink. The decrease in water content and the increase in polyol content synergistically enhanced the size exclusion effect of polyols [46,47], resulting in a reduction in recovery. A clear process of viscosity recovery appeared with the increase of polyols, which might be related to the increase in viscosity of polyols, which was consistent with the previous conclusion. However, sorbitol recovery was significantly lower than xylitol and erythritol.

### 3.6. Rheological Characteristics of Inks Governing Self-Supporting Stage

**Figure 5:**
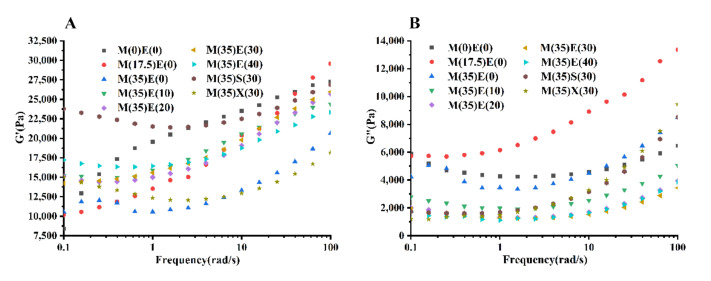

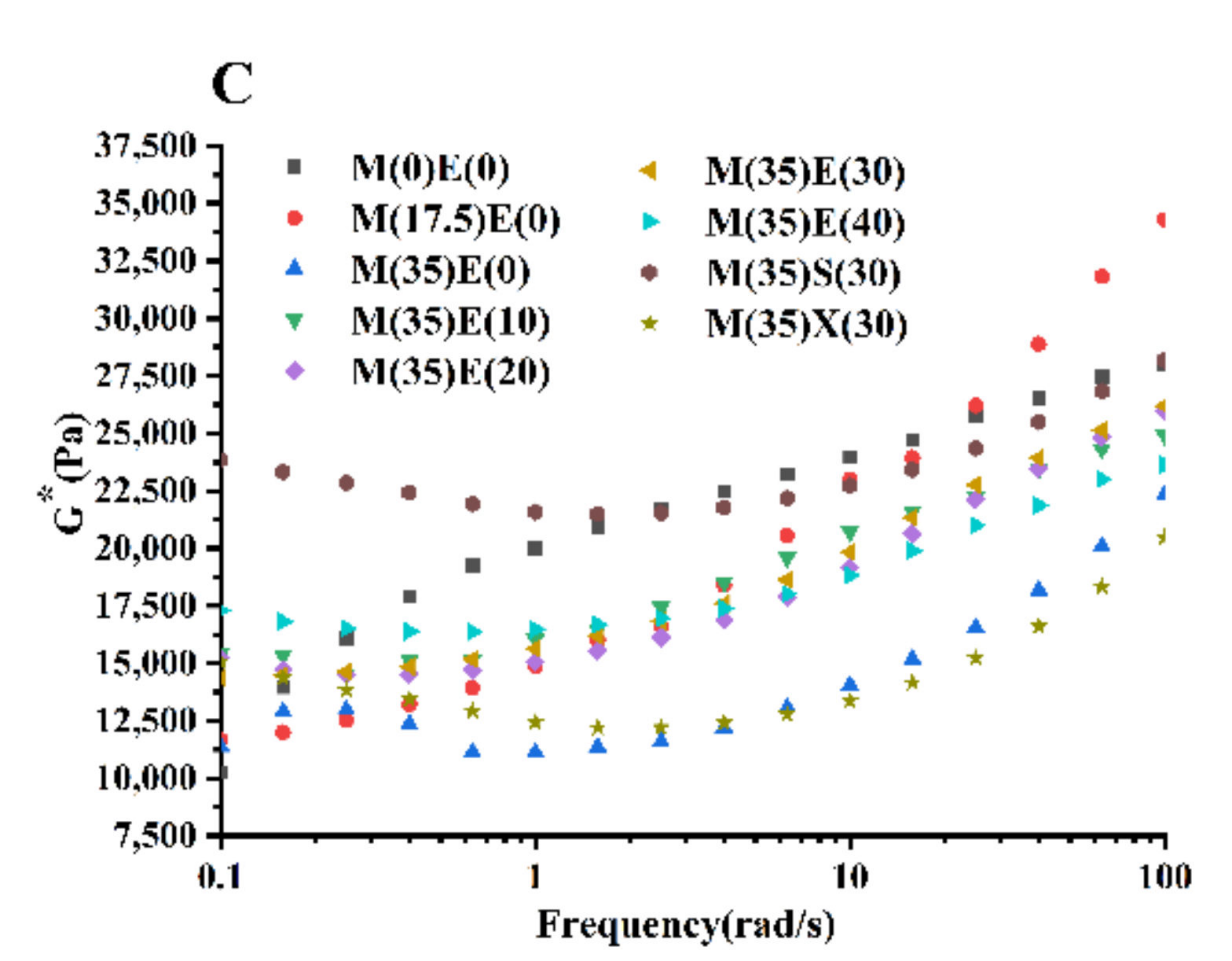
(**A**) Viscoelasticity properties of different inks within frequency of 1–100 rad/s conducted at room temperature 25 °C. (**A**) Elastic modulus. (**B**) Viscous modulus. (**C**) Complex modulus.

Figure 5 shows the variation curves of G′, G″, and G* with angular frequency in each ink. G* represents the solid-like property and reflects the ability of compressive deformation and mechanical strength in the process of self-supporting [25,35,36]. Inks with sufficient mechanical strength will exhibit excellent self-supporting behavior [43]. The gel system without polyols had obvious frequency dependence, which was very obvious at low frequency, which indicates that it had no stable solid properties [37,41]. With the addition of polyols, solid-like behavior (G′ with nearly zero slope) [48] is observed in Figure 5A with the addition of polyols at low frequencies. It is convenient that the gel exhibits this behavior at low frequencies, since this allows it to support its own structure after being deposited on the printing bed. According to Kasapis et al. [49], ink with solid-like behavior will have a better printing performance, that is why with the addition of polyols gummy gel would have a better performance. The G″ reflects the liquid properties of the gel, and the gel without polyol had the highest loss modulus at low frequencies, which was related to its high-water content and poor solid properties [37,41]. Figure 5B shows G″ gradually decreased with the addition of polyols, which may be due to excess polyols inhibiting the hydration of gels. Solid-like behavior also showed at low frequencies. This is consistent with the G′ analysis. Figure 5C shows G* indicates that all inks have a good self-supporting characteristic. It can be considered that gummy ink has gel-like behavior and relatively robust network structure [50]. However, it is also reported that the gel system shows more polyol characteristics with more sweetener content [27]. Compared with sorbitol and xylitol inks, erythritol inks change less at low frequencies—all the inks have better self-support performance.

It is worth mentioning that due to the limitations of the equipment, it is not possible for the inks to reach a steady state at such a short time, the G′, G″, and G* at test temperature (25 °C) might not really reflect the deformation resistance performance, but they can still give us useful information about ink’s self-supporting behaviors.

### 3.7. Textural Properties

**Figure 6:**
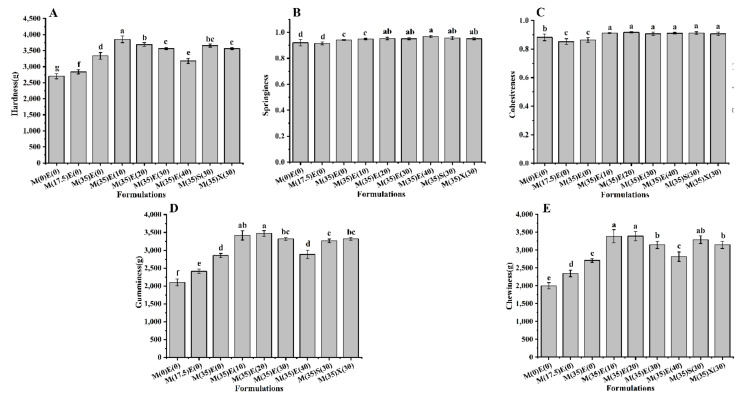
Textural properties with different formulation inks. (**A**) Hardness; (**B**) Springiness; (**C**) Cohesiveness; (**D**) Gumminess; (**E**) Chewiness. Different lowercase letters denote significant differences (*p* < 0.05).

TPA is considered an effective descriptor of the hydrophilic colloidal properties of polysaccharides [51]. Five parameters, including hardness, elasticity, viscosity, cohesiveness, and chewiness were selected to evaluate the effect of polyols on gel soft sweets content and describe the sensory characteristics of printed gummies. All the gummies contained the same gel content, and the hardness increased gradually with the addition of maltitol (Figure 6A). After the addition of 10 g erythritol, the hardness reached a maximum value of 3853 g, as erythritol was continued to be added, the strength of the gel network decreased gradually—the hardness was only 3181 g when adding 40 g erythritol. This result is due to the fact that a large amount of polyols would affect the gel link to decrease its network structure [22].

With the addition of polyol, there is no obvious difference in the springiness and cohesiveness of the ink (Figure 6B, C). The springiness of the ink with 17.5 g maltitol and the ink without polyol were the lowest, about 0.915, and the springiness of the ink with 40 g erythritol was the highest—0.968. The cohesiveness of the ink with 17.5 g maltitol was the lowest, 0.852, slightly lower than ink without polyol and the cohesiveness of the ink with 20 g erythritol was the highest, 0.911. Gumminess and chewiness also show a change trend of increase first and then decrease this is similar to the change in hardness (Figure 6D,E), the actual mouth feel of gummies are related to gumminess and chewiness, the addition of polyols can not only increase the sweetness, but also result in the mouth feel of gummies to become more suitable for consumption.

Whereas inks with xylitol, sorbitol, and the 30 g erythritol protocol have similar textural properties, as previously reacted for by Textural properties, sorbitol and xylitol have higher hardness, gumminess, and chewiness, while springiness and cohesiveness are not significantly different. This is more due to the characteristics of different polyols.

### 3.8. FTIR on Simulation System

**Figure 7:**
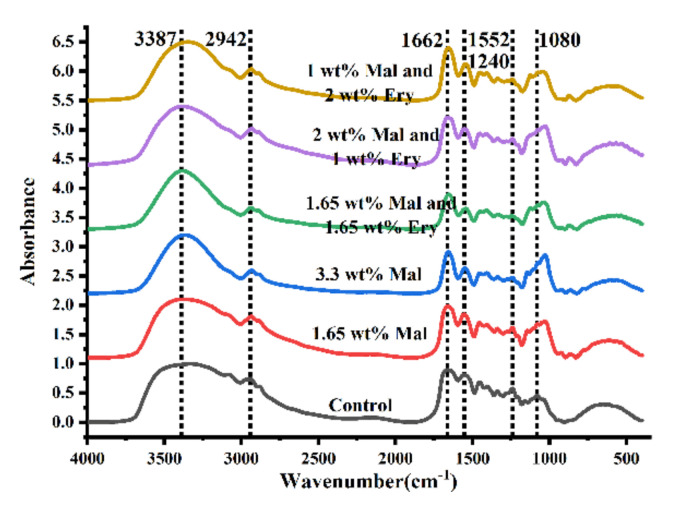
The FTIR spectra of maltitol, erythritol, gelatin and low acyl gellan gel with different maltitol and erythritol content.

The rheological results showed that polyols affected the rheological properties of gummies through hydrogen bond and steric hindrance. Therefore, by maintaining the fixed ratio of gelatin and low acyl gellan, a low concentration polyol system was set up to observe the absorption peak position and to explore the effect of polyol addition on the gel structure. In the experiment, a gel containing no polyols was set as a control. The spectra revealed that the ink maintained the typical structure of gelatin with low acyl gellan (Figure 7). Six regions of the spectrum (amides A, B, I, II, and III and the assigned region) were investigated experimentally. The amide A band of all the samples is around 3387 cm^−1^, close to the free N-H and H bond stretching frequencies, but it tends to shift toward lower frequencies with the addition of polyols. Giménez et al. reported that polyols mainly formed hydrogen bonds with gels [52]. Amide B near 2942 cm^−1^, reflecting C-H single bond antisymmetric and symmetric stretching, had no noticeable change for all additions. The amide I band was found to be related to the secondary structure of proteins; it is located at 1662 cm^−1^ with no significant shift for all samples. The amide II bands at (1552 cm^−1^, 1453cm^−1^, 1339cm^−1^) decreased and probably shifted to a lower wavenumber with the addition of polyols, which has been suggested to reflect hydration [53]. While the amide III band was located at about 1240 cm^−1^, which was less and less obvious with the addition of polyols. The absorption peak at about 1080 cm^−1^ can be attributed to stretching vibration of C–OH side groups and band vibration of C–O–C glycosides [53,54,55].

The area in the wavelength range 1700−1600 cm^−1^ can be used to examine the secondary structure of gelatin: β-sheet is represented by the peak at 1610−1642 cm^−1^; random coil is represented by the peak at 1642−1650 cm^−1^; α-helix is represented by the peak at 1650−1660 cm^−1^; β-turn is represented by the peak at 1660−1680 cm^−1^; and β-antiparallel is represented by the peak at 1680−1700 cm^−1^ [56]. Table 3 showed the results after curve fitting and deconvolution, with no significant changes in β-sheet, random coil, α-helix, β-turn, and β-antiparallel. However, this necessitates caution because the inclusion of polyols enhanced the β-sheets, hence boosting the gel characteristics [57,58]. Sugar, according to Sow et al., had a particular effect on the triple helix concentration of gelatin via forming hydrogen bonds [59,60].

Polyols did not change the main gel network formed by gelatin with low acyl gellan and did not generate new functional groups. Both gels and polyols contain hydroxyl groups, and their mixing and subsequent gelation did not lead to generation or disappearance of functional groups, but may easily have formed intramolecular or intermolecular hydrogen bonds [30]. This could have an impact on protein structure as well. The inclusion of polyols boosted the gel’s viscosity and hardness, according to the research. The presence of hydrogel in the ink is unmistakable. When the polyol concentration is too high, it is unable to create a stable hydrogen bond with the gel, resulting in a gradual loss in hardness.

### 3.9. SEM on Simulation System

**Figure 8:**
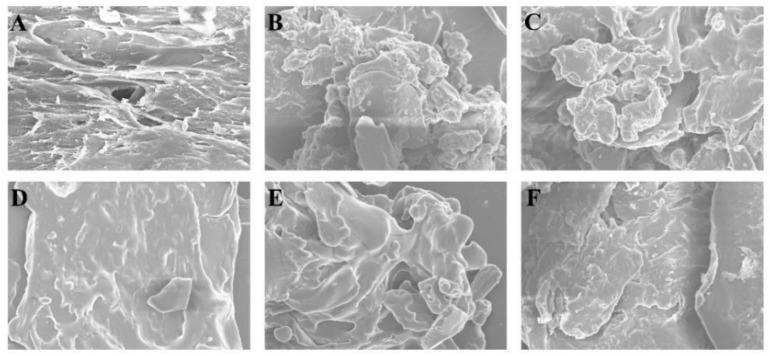
The microstructures of the maltitol, erythritol, gelatin, and low acyl gellan gel with different maltitol and erythritol content. The magnifications of each group images are set as 1000×. (**A**) control group. (**B**) Contain with 1.65 wt% Mal. (**C**) Contain with 3.3 wt% Mal. (**D**) Contain with 1.65 wt% Mal and 1.65 wt% Ery. (**E**) Contain with 2 wt% Mal and 1 wt% Ery. (**F**) Contain with 1 wt% Mal and 2 wt% Ery.

In order to understand the influence of polyols on gel system intuitively, the ratio of gelatin to low acyl gellan was maintained at 10:1 to prepared low concentration ink system and explore the effect of polyols on the microstructure of gel system. The Figure 8 shows the effects of adding maltitol and erythritol to the mixed gel. The SEM of the blend of gelatin and low acyl gellan can be seen from the diagram. The synergistic impact of low acyl gellan and gelatin can build a dense gel network [20], the pores structure is thought to be generated by hundreds of spiral structures layered on top of each other [50], but the surface is not very smooth. The holes become harder to identify as maltitol is added, and the surface gets flatter, although aggregates remain. This could be owing to maltol’s larger molecular weight, which allows it to offer more -OH, as well as polyol’s favorable hydration in the bulk aqueous phase, which allows it to be excluded from the gel’s surrounds. The gel–water interactions were diminished as a result, and the gel strands switched from self-association to association with their neighbors [22]. Polyol binding to gel helices via an intermolecular cross-linked hydrogen connection between gel and polyol aided in the aggregation of surrounding junctions into large junctions or aggregates [61]. After erythritol is added, the aggregation is reduced, and the surface becomes smoother. This may be one of the reasons why the extrusion properties improved after adding erythritol to the ink, indicating smoother changes. However, if polyol content continues to rise, either polyol–gel or the connection between polyols and gel aggregates will become weak. The gel will eventually deteriorate, resulting in a loss of textural qualities. The entire system will exhibit more polyol properties, such as increased viscosity and improved extrusion performance.

## 4. Conclusions

The confectionary sector now has a better answer thanks to 3D printing technology. The ink provided the best printing effect when the moisture content was 30% in the experiment, and when 30 g erythritol was added. Rheological research found that the ink exhibits typical temperature sensitive shear-thinning behavior at this time, as well as good viscosity at high shear rates and low yield stress, and, where 3ITT studies show, leads to the best recovery. The influence of polyols on 3D printing of soft sugar gels is mostly apparent in the way that polyols improve the gel behavior through hydrogen bonding and the steric hindrance effect created after polyol incompatibility with gels, according to rheological studies. With increasing polyol concentration, the former intensifies and provides a steric hindrance effect, increasingly dominating the ink’s rheological features. The printing properties of sorbitol and xylitol inks support this conclusion: sorbitol has the biggest molecular weight and can create more hydrogen bonds, and its yield stress, gel temperature, and viscosity are all much higher than those of erythritol inks, although xylitol is similar. Microanalysis of the simulated systems also confirmed that polyols improve gel properties by strengthening the hydrogen bonding connection, whilst the inclusion of low viscosity polyols smoothens the ink and reduces the formation of agglomerates. When 3D printing soft candies, the sweeteners with the lowest viscosity performance are used, and the ink has a higher printing performance. The gummy gel is a complicated system, and our investigations showed that by tweaking the ink formulation, it may be made more suitable for 3D printing. This study will serve as a guide for creating bespoke 3D printing gel fudge with additional polyols as sweeteners. This study on the effects of polyols on the qualities of 3D printed food inks may bring further suggestions for 3D printed food ink preparation.

## Figures and Tables

**Table 1:** Composition of the printable gummy ink formula and final water content.

Serial Number	Water (mL)	Gelatin (g)	Low Acyl Gellan (g)	Maltitol (g)	Erythritol (g)	Sorbitol (g)	Xylitol (g)	Water Content (%)
M(0)E(0)	60	14	1.4	0	0	0	0	77.8
M(17.5)E(0)	60	14	1.4	17.5	0	0	0	44.4
M(35)E(0)	60	14	1.4	35	0	0	0	39.6
M(52.5E(0)	60	14	1.4	52.5	0	0	0	37.2
M(35)E(10)	60	14	1.4	35	10	0	0	35.3
M(35)E(20)	60	14	1.4	35	20	0	0	33.6
M(35)E(30)	60	14	1.4	35	30	0	0	30
M(35)E(40)	60	14	1.4	35	40	0	0	28.1
M(35)S(30)	60	14	1.4	35	0	30	0	30
M(35)X(30)	60	14	1.4	35	0	0	30	30

**Table 2:** Formulation of low concentration polyol gel for microanalysis.

Serial Number	Water (mL)	Gelatin (g)	Low Acyl Gellan (g)	Maltitol (g)	Erythritol (g)
1	100	3	0.3	0	0
2	100	3	0.3	1.65	0
3	100	3	0.3	3.3	0
4	100	3	0.3	1.65	1.65
5	100	3	0.3	2	1
6	100	3	0.3	1	2

**Table 3:** Secondary structure percentage (%) analysis of different maltitol and erythritol content gel by analyzing the areas of 1600–1700 cm^−1^ in FTIR spectra.

Sample	β-Sheet	Random Coil	α-Helix	β-Turn	β-Antiparallel
Control	34.82 ± 3.40 ^a^	10.21 ± 0.60 ^b^	10.30 ± 0.33 ^a^	30.50 ± 2.45 ^a^	14.17 ± 0.20 ^a^
1.65% M	35.03 ± 3.23 ^a^	10.27 ± 0.54 ^a^	10.52 ± 0.29 ^a^	30.68 ± 3.53 ^a^	13.49 ± 0.32 ^ab^
3.3% M	35.23 ± 2.91 ^a^	10.87 ± 0.46 ^a^	10.95 ± 0.34 ^a^	30.32 ± 3.56 ^a^	12.63 ± 0.31 ^c^
1.65% M + 1.65% E	35.34 ± 2.63 ^a^	10.95 ± 0.51 ^a^	10.91 ± 0.36 ^a^	30.66 ± 3.70 ^a^	12.14 ± 0.53 ^c^
2% M + 1%E	35.13 ± 2.87 ^a^	10.57 ± 0.49 ^a^	10.78 ± 0.30 ^a^	30.66 ± 5.20 ^a^	12.85 ± 0.64 ^bc^
1% E + 2%M	35.02 ± 3.72 ^a^	10.91 ± 0.28 ^a^	10.73 ± 0.35 ^a^	30.68 ± 4.23 ^a^	12.39 ± 0.35 ^c^

1.65% M, 3.3% M, 1.65% M + 1.65% E, 2% M + 1% E, 1% M + 2% M: gelatin and low acyl gellan gel with different maltitol and erythritol content. For the same column, different superscripts indicate significant differences (*p* < 0.05).

## Data Availability

The data presented in this study are available on request from the corresponding author.

## Supplementary Materials

The following supporting information can be downloaded at: https://www.mdpi.com/article/10.3390/foods11060874/s1, Figure S1. Temperature ramp tests with a cooling rate of 1 °C/min for ink of different polyol formulations; Figure S2. Printing photos of control group ink (M(0)E(0)).
