# Supplementary material for: Making Polyol Gummies by 3D Printing: Effect of Polyols on 3D Printing Characteristics
Source: Foods. 2022 Mar 18;11(6):874. doi: 10.3390/foods11060874 (PMC8950482; doi:10.3390/foods11060874)
Supplement: Supplementary file 1 [file foods-11-00874-s001.zip › foods-1616987-supplementary.pdf]

foods

Supplementary Materials

Making Polyol Gummies by 3D Printing: Effect of Polyols on 3D Printing Characteristics

Hao Le [1], Xiaorui Wang [1], Yabo Wei [1], Yunfeng Zhao [1], Jian Zhang [1,*] and Lianfu Zhang [2,*]

[1] Food College, Shihezi University, Shihezi 832003, China; 20192111005@stu.shzu.edu.cn (H.L.); wangxiaorui0923@163.com (X.W.); 18935813163@163.com (Y.W.); yunfeng@shzu.com.edu.cn (Y.Z.)
[2] School of Food Science and Technology, Jiangnan University, Wuxi 214122, China
* Correspondence: zhangjian0411@163.com (J.Z.); lianfu@jiangnan.edu.cn (L.Z.); Tel.: +86-189-9773-1657 (J.Z.); +86-138-1219-2381 (L.Z.)

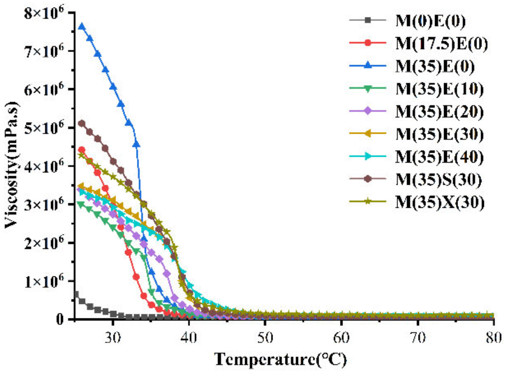

Figure S1. Temperature ramp tests with a cooling rate of 1 °C/min for ink of different polyol formulations.

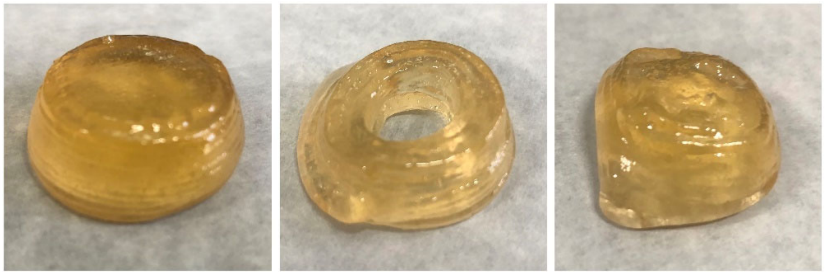

Figure S2. Printing photos of control group ink (M(0)E(0)).
